# Drought Tolerance in *Pinus halepensis* Seed Sources As Identified by Distinctive Physiological and Molecular Markers

**DOI:** 10.3389/fpls.2017.01202

**Published:** 2017-07-24

**Authors:** Khaled Taïbi, Antonio D. del Campo, Alberto Vilagrosa, José M. Bellés, María Pilar López-Gresa, Davinia Pla, Juan J. Calvete, José M. López-Nicolás, José M. Mulet

**Affiliations:** ^1^Faculty of Natural Sciences and Life, Ibn Khaldoun University Tiaret, Algeria; ^2^Re-ForeST, Research Institute of Water and Environmental Engineering, Universitat Politècnica de València Valencia, Spain; ^3^Instituto de Biología Molecular y Celular de Plantas, Universitat Politècnica de València – Consejo Superior de Investigaciones Científicas Valencia, Spain; ^4^Fundación Centro de Estudios Ambientales del Mediterráneo, Joint Research Unit University of Alicante – CEAM, University of Alicante Alicante, Spain; ^5^Instituto de Biomedicina de Valencia, Consejo Superior de Investigaciones Científicas Valencia, Spain; ^6^Departamento de Bioquímica y Biología Molecular-A, Facultad de Biología, Universidad de Murcia Murcia, Spain

**Keywords:** *Pinus halepensis*, Aleppo pine, drought tolerance, physiological response, soluble sugars, free amino acids, plant proteomics, glutathione

## Abstract

Drought is one of the main constraints determining forest species growth, survival and productivity, and therefore one of the main limitations for reforestation or afforestation. The aim of this study is to characterize the drought response at the physiological and molecular level of different *Pinus halepensis* (common name Aleppo pine) seed sources, previously characterized in field trials as drought-sensitive or drought-tolerant. This approach aims to identify different traits capable of predicting the ability of formerly uncharacterized seedlings to cope with drought stress. Gas-exchange, water potential, photosynthetic pigments, soluble sugars, free amino acids, glutathione and proteomic analyses were carried out on control and drought-stressed seedlings in greenhouse conditions. Gas-exchange determinations were also assessed in field-planted seedlings in order to validate the greenhouse experimental conditions. Drought-tolerant seed sources presented higher values of photosynthetic rates, water use efficiency, photosynthetic pigments and soluble carbohydrates concentrations. We observed the same pattern of variation of photosynthesis rate and maximal efficiency of PSII in field. Interestingly drought-tolerant seed sources exhibited increased levels of glutathione, methionine and cysteine. The proteomic profile of drought tolerant seedlings identified two heat shock proteins and an enzyme related to methionine biosynthesis that were not present in drought sensitive seedlings, pointing to the synthesis of sulfur amino acids as a limiting factor for drought tolerance in *Pinus halepensis*. Our results established physiological and molecular traits useful as distinctive markers to predict drought tolerance in *Pinus halepensis* provenances that could be reliably used in reforestation programs in drought prone areas.

## Introduction

In the context of climate change, the fate of many forest ecosystems depends on their adaptation to such changes (Chen and Jiang, 2010). Interspecific variation in key functional traits along environmental gradients can explain adaptive patterns related to environmental cues (Sánchez-Gómez et al., 2010). This variation can happen at an intraspecific level, which would explain adaptive patterns among different populations throughout the geographical range of individual species (Taïbi et al., 2014, 2015). Hence, characterization of this variation within forest tree species is relevant to understand the interaction and significance of evolutionary forces, and to carry out appropriate, genetically based breeding programs (White et al., 2007). Traditionally, for reforestation and ecological restoration, the common strategy was based on the use of local genotypes or genotypes previously represented. One defect of this approach is that it does not consider forest migration and changes in environmental factors, which may lead to failure in reforestation programs and to an unnecessary waste of resources and time (Williams and Dumroese, 2013).

Regional-scale forest mortality worldwide has been associated to drought and predicted climate change is expected to aggravate the negative impact of extreme drought events (McDowell, 2011). In light of climate change predictions in the Mediterranean region, it is unclear how *Pinus halepensis* (Aleppo pine), an important forest tree in this region, will adapt and persist in reforestation carried out in the most limiting conditions for this species in the future (Maestre and Cortina, 2004). Furthermore, drought is demonstrated to have strong implications on the distribution of forest tree species and is able to limit their geographical distribution (Taïbi et al., 2014).

Marked differences in terms of growth, survival and other adaptive attributes have been reported for different *Pinus halepensis* seed sources originating from most of its climatic and ecologic range in Spain and tested under drought conditions in different trial sites (Taïbi et al., 2014, 2015). These studies revealed the selective role of climate variables in determining each populations’ fitness within this species. However, to explain and understand the mechanisms underlying such intraspecific variability, seedling establishment must be analyzed at the molecular and physiological levels (Margolis and Brand, 1990; Grossnickle, 2012). Comparing the physiological and molecular responses among drought-tolerant and drought-sensitive populations and the identification of differential traits can be a useful tool to predict the response of uncharacterized populations before planting them in drought prone areas. This information can be valuable for the conservation of genetic resources, and can facilitate successful assisted migration programs (Taïbi et al., 2014). In fact, the seedling stage is a critical part of the tree’s life cycle during which the plant is highly susceptibility to resource limitations that may affect survival, establishment and growth, and thus preventing the success of many reforestation programs (Leck et al., 2008).

Studies addressing integrated ecophysiological response (survival, growth, physiological and molecular responses) of forest trees under stressed conditions could generate important data. However, to the authors’ knowledge, no study has incorporated *Pinus halepensis* intraspecific variability as an explicit factor to study such integrated response in plantation establishment. At the physiological level, *Pinus halepensis* is known to prevent water stress damage by stomatal closure before strong changes occur in needle water potential (Baquedano and Castillo, 2006) following an isohydric strategy (Klein et al., 2012). As a consequence of stomatal closure, carbon assimilation can be completely inhibited (Martínez-Ferri et al., 2000), thereby increasing the risk of oxidative stress. The impairment of photosynthetic carbon assimilation by stomatal closure affects the metabolic balance in plants (Pinheiro et al., 2011). Soluble sugars are among the most drought-responsive metabolites, increasing due to starch hydrolysis or impairment of starch production (Rodríguez-Calcerrada et al., 2011). In fact, soluble sugars correlate negatively with photosynthesis (Franck et al., 2006). Moreover, the maintenance of osmotic adjustment is important for normal cell activity and survival (Bartlett et al., 2012). In many forest species, organic solutes, specifically soluble carbohydrates and free amino acids, are the principal compounds involved in osmotic adjustment (Patakas et al., 2002). At the molecular level, most of these responses depend on proteins, therefore comparison of the protein profile of tolerant and sensitive populations can provide valuable information to predict the performance of a given genotype under drought conditions (Hu et al., 2012).

Previously, we have identified drought tolerant and drought sensitive seed sources of *Pinus halepensis* based on a 4-year field experiment (Taïbi et al., 2014, 2015). In the present work, we characterize the physiological and molecular responses of different *Pinus halepensis* seed sources under controlled drought stress. Studies at the molecular level require the use of controlled greenhouse conditions, given that in the field there are many variables such as the presence of pathogens, different light exposure, wound stress caused by strong wind, rain or insect attack or mechanical stimulation that can differentially affect several plants and therefore flaw the results. The objective of this work is to characterize the response of different *Pinus halepensis* seedlings (drought tolerant vs. drought sensitive) at the physiological, biochemical and molecular levels under controlled drought stress to identify patterns and/or features between seed sources previously classified as tolerant or sensitive (Taïbi et al., 2014). We expect that the generated data can be useful to predict if seed sources of *Pinus halepensis* for which no field trials have been carried out will be suitable for planting in areas with either current or predicted drought conditions.

## Materials and Methods

### Plant Material

This study was performed using four *Pinus halepensis* seed sources, covering most of the climatic and ecological regions of the species natural range in Spain and representing considerable phenotypic variation (Taïbi et al., 2014). These seeds have been tested previously in field provenance trials over 3 years (1 year in the nursery+3 years in the field) for survival and growth (height and stem diameter) under drought stress. During the summer of the second year after planting, additional physiological measurements were taken at the trial sites and will be used here as an independent data set to assess seed source response. Seed sources of ‘Levante Interior’ (Lev), ‘La Mancha’ (Man) were considered drought-tolerant while those of ‘Ibérico Aragonés’ (Arg) and ‘Alcarria’ (Alc) were considered drought-sensitive based on the results obtained in Taïbi et al. (2014, 2015).

### Experimental Conditions and Treatments

Seedlings were grown following common procedures reported in the literature for this species in Forespot© 300 containers. Each 16-cm-deep plastic tray consists of 54 cells providing a density of 360 seedlings m^-2^ (Villar-Salvador et al., 2004) filled with a mixture of sphagnum peat vermiculite-pine bark (3:1:1 v/v) and arranged in a complete random block design with six blocks where the different seed sources were randomized within the block.

Seedlings were grown in a growth chamber under controlled conditions set at a 24°C/16°C day/night temperatures, relative humidity at 70% and a photoperiod of 16 h (200 μmol m^-2^ s^-1^). Seedlings were watered to full capacity every 2 days alternatively twice by water and once by complete Hoagland’s nutrient solution (Hoagland and Arnon, 1950) containing all essential macro and micro-nutrients.

After 25 weeks of growth, healthy plants of similar size from each seed source, accounting for 15 replicates per seed source, were randomly assigned to control and drought treatment; control seedlings were irrigated every 2 days whereas drought conditions were applied through withholding water until seedling weight (plant and container) was reduced to 60% of their initial weight (to a predawn water potential that was 60% of the loss of turgency point), thus avoiding catastrophic xylem cavitation and deleterious associated effects. Measurements were carried out at the end of the duration of the drought treatment after 3 weeks (Vilagrosa et al., 2003; Villar-Salvador et al., 2004).

### Measurements

#### Water Potential

Seedling water potential (Ψ_w_, MPa) was measured with a Scholander-type pressure pump (model PMS-1000, PMS Instruments, Corvallis, OR, United States) on five seedlings selected randomly from each seed source per treatment. Measures were carried out at predawn.

#### Photosynthetic Gas Exchange and Chlorophyll Fluorescence

Instantaneous determinations of net CO_2_ assimilation (*P*_n_, μmol CO_2_ m^-2^ s^-1^), stomatal conductance (*g*_s_, mol m^-2^ s^-1^), transpiration *E* (mmol H_2_O m^-2^ s^-1^), and instantaneous water use efficiency (WUE_inst_; μmol CO_2_ mmol^-1^ H_2_O) calculated as assimilation divided by transpiration *P*_n_/*E*, were determined in five seedlings per seed source per treatment using a portable photosynthesis open-system (Model LI-6400, LI-COR Biosciences Inc., Lincoln, NE, United States). Gas exchange variables were also estimated under conditions of saturating light (1500 μmol photon m^-2^ s^-1^), 25°C and environmental CO_2_ (390 μmol mol^-1^ CO_2_) maintaining the relative humidity in the chamber at approximately 55 ± 5%. All gas-exchange measurements were expressed as a function of needle-projected area.

Maximal photochemical efficiency of PSII (*F*v*/F*m) was determined at predawn using a chlorophyll fluorometer (PAM 2000, Walz, Effedrich, Germany). Φ_PSII_ (quantum yield of non-cyclic electron transport) was estimated as (Fm′–Fs′)/Fm′ under steady-state conditions of illumination. It was determined early in the morning by using an open gas exchange system (LI-6400; LI-COR, Inc., Lincoln, NE, United States) with an integrated fluorescence chamber (LI-6400-40 leaf chamber fluorometer; LI-COR). Φ_PSII_ was determined in the same set of needles used for the gas exchange analysis. Maximal efficiency of PSII and Φ_PSII_ were calculated according to Maxwell and Johnson (2008).

Transpiration, net photosynthesis and maximal efficiency of PSII were also measured on the trial sites ‘La Hunde’ (used as a control, representing the core habitat for the species) and in ‘Granja d’Escarp’ trial site (used as a dry site, representing a marginal dry habitat of the species). A complete description of these sites can be found in Taïbi et al. (2014). These measurements were used as a control to compare with the greenhouse conditions.

Needle transpiration (*E*) and photosynthesis rate (*P*n) were measured in field conditions on five plants per seed source per site (randomly selected in one block) using an LCpro+ Portable Photosynthesis System with a leaf chamber (ADC Bioscientific Ltd., Hoddesdon, Hertz, EN11 0DB) with a light unit; ambient CO_2_ was set at 390 ppm, air humidity at 60–70%, temperature at 25°C and photosynthetic photon flux density (PPFD) at 800 μmol m^-2^ s^-1^. *In vivo* measurements were performed on intact needles with no visible symptoms of damage. Analysis of leaf area, was performed with a numeric scanner connected to the WinRhizo software (Regent Instruments Inc., Quebec City, QC, Canada). Additionally, the efficiency of photochemical reactions driving photosynthesis was assessed by chlorophyll fluorescence measurements. Minimal fluorescence (*F0*) yield was obtained upon excitation with a weak measuring beam from a pulse light-emitting diode, while maximal fluorescence yield (*F*m) was determined after exposure to a 0.8 s saturating pulse [>10,000 μmol (photon) m^-2^ s^-1^] of white light. The maximal efficiency of PSII was then estimated as the ratio of variable to maximal fluorescence (Fv/Fm = (Fm–Fo)/Fm; Schreiber et al., 1994).

### Metabolite Analysis

Chlorophyll a (Chl a), chlorophyll b (Chl b), and carotenoids (Car) concentrations were determined spectrophotometrically using the Lichtenthaler method (Lichtenthaler, 1987). Non-structural carbohydrates (NSC) were determined by grinding 0.2 g of needles (fresh weight) in liquid nitrogen with a mortar and pestle, and then the homogenized powder was resuspended in 1 mL water and measured as described in Fayos et al. (2006). Specifically, the samples were incubated at 95°C for 10 min, cooled on ice and centrifuged at 4°C for 5 min to remove debris. The supernatants were filtered through Sep-Pak Plus C-18 solid phase cartridges (Waters). The soluble sugar fraction (mono and oligosaccharides) was separated by chromatography in a Waters 1525 HPLC system equipped with an evaporative light scattering detector (2424 ELSD). Aliquots (20 μl) were injected into the column ProntoSil 120-amino 3 μm (125 mm × 4.6 mm i.d.) with a Waters 717 autosampler. Elution was carried out at room temperature under isocratic conditions using a mixture of acetonitrile (J.T. Baker) and H_2_O (Milli-Q Millipore) (85:15) at a flow rate of 1 mL/min during 25 min. The conditions of the light scattering detector were the following: gain 75, data rate 1 pps, nebulizer heating 60%, drift tube 50°C, and gas pressure 40 psi. Sugars were quantified with the Waters Empower software using glucose, fructose, sucrose, and sorbitol standards calibration curves. Glutathione (GSH) and free amino acids were extracted from 2 grams of needles according to the method described in Mulet et al. (2004). In brief, plant material was pooled and homogenized in liquid nitrogen. Each pooled sample (0.10 g of FW) was heated 12 min at 95°C in 2% isocitrate buffer (pH 2 with HCl). 1/10 dilutions of these extractions were injected in a Beckman Gold amino acid automatic analyser. The analysis was carried out following the protocol provided by the manufacturer, using a sodium citrate system and ninhydrin for detection.

### Extraction, Separation and Identification of Proteins by LC-MS/MS Analysis

One gram of plant material obtained from the seedlings growth in chamber (root or needle) was ground to powder with liquid nitrogen with the addition of 0.1 g of polyvinylpolypyrrolidone (PVPP) and extracted with 10 mL of extraction buffer (5% sucrose, 4% SDS and 5% 2-ME) for 10 min at room temperature with gentle stirring, followed by centrifugation at 10.000 *g*. The clear supernatant was heated at 100°C for 3 min and then cooled at room temperature. Proteins were precipitated by adding eight volumes of cold acetone. After at least 1 h at -20°C, the mixture was centrifuged at 10.000 *g*. The pellet was re-suspended in 5 mL of extraction buffer and centrifuged at 10.000 *g* and washed twice with 80% cold acetone, and precipitated by adding four volumes of cold acetone, and was then lyophilized and stored at 0°C (He et al., 2007). The concentrations of extracted proteins were determined using the Bradford method (Bradford, 1976). Proteins were separated by denaturing polyacrylamide gel electrophoresis (SDS–PAGE) using a 5–20% gradient gel. Gels were visualized by staining with Coomassie Brilliant Blue with some modifications as described in Mulet et al. (2006). Rubisco content was used as a visual loading control, as we did not found significant differences in rubisco content among different species. Proteins were identified using standard protein mass spectrometry techniques.

### Statistical Analysis

Data were subjected to analysis of variance with tolerance/sensitivity and seed sources as variables, seed source was nested to tolerance/sensitivity to determine differences among drought-tolerant and drought-sensitive seed sources separately, under watered conditions, and then under drought stressed conditions. Additional analysis of variance was carried out to determine significant differences between means at *P* < 0.05 level. Homogeneous groups were separated using the Duncan’s test. In all cases, data were examined for normality and homogeneity of variances and assessed for any violations of assumptions using the Duncan’s test (Duncan, 1955).

## Results

### Water Potential and Gas Exchange

**FIGURE 1:**
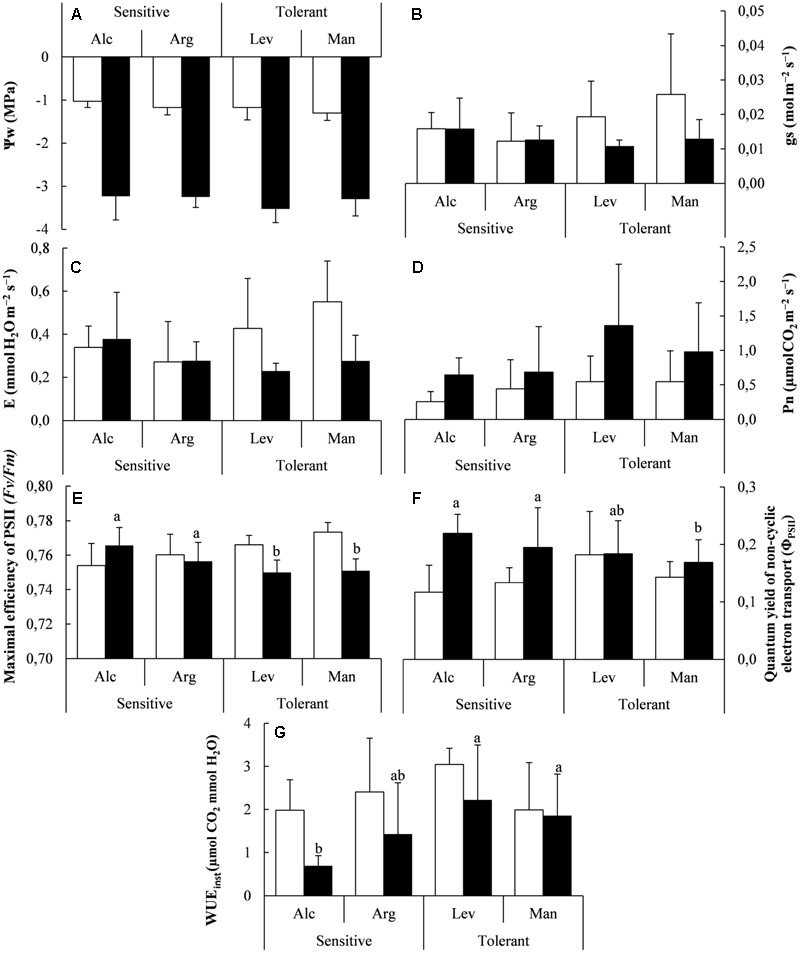
Water potential, gas exchange and photosynthesis measurements under greenhouse conditions. Water potential (Ψw) **(A)**, stomatal conductance (*g*_s_) **(B)**, transpiration (E) **(C)**, Net photosynthesis (*P*n) **(D)**, Maximal efficiency of PSII **(E)**, quantum yield of non-cyclic electron transport (Φ_PSII_) **(F)** and instantaneous water use efficiency (WUE_inst_) **(G)** of drought-sensitive and drought-tolerant seed sources under watered (white bars) and drought stressed (black bars) treatments. The letters above the bars marks the significant difference among the drought-stressed seed sources following the *post hoc* Duncan’s test. Scale bars are mean +SE, being the number of samples *n* = 15 for Ψw and *n* = 5 for the other variables.

The first aim of our study was to confirm if our greenhouse conditions were indeed reflecting real drought conditions and to observe if there were any differences among seedlings from different provenances. Under controlled drought stress, water potential (Ψ_w_) decreased significantly by 3.5-fold in both drought-tolerant and drought-sensitive seed sources (**Figure 1A**), indicating that the plants were experiencing drought stress. We also observed that stomatal conductance (*gs*) and transpiration (*E*) showed a higher tendency in drought tolerant seedlings under normal conditions (**Figures 1B,C**). In this sense, only net photosynthesis (*P*_n_) was statistically significant between tolerant and sensitive seed sources when analyzed globally (independently of the watering level) but no differences can be found among means values for each seed source. The photosynthetic rates were about twofold higher in the drought-tolerant seed sources when compared among sensitive/tolerant origins (that is averaged values about 0.41 ± 0.06 and 0.89 ± 0.16 μmol CO_2_ m^-2^ s^-1^ for sensitive and tolerant, respectively; **Figure 1D**). No statistical differences were observed between control values and drought stressed ones indicating that the response in terms of *P*n was similar in both conditions.

**FIGURE 2:**
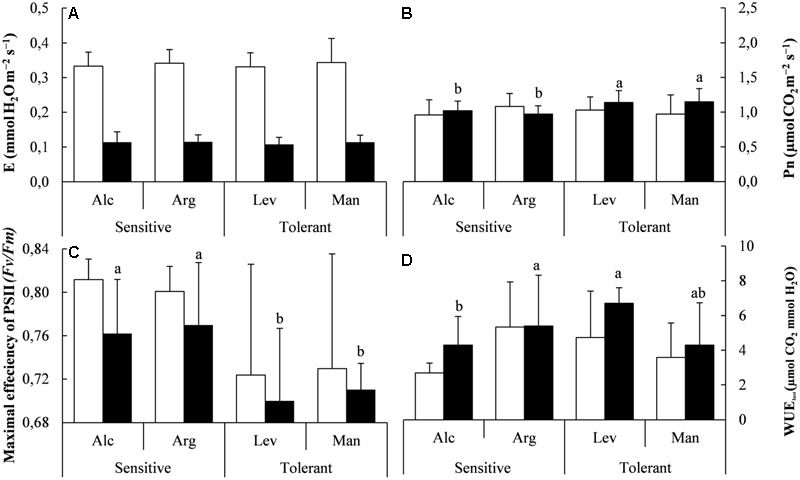
Gas exchange, photosynthesis measurements and water use efficiency under field conditions. Transpiration (E) **(A)**, Net photosynthesis (*P*n) **(B)**, Maximal efficiency of PSII **(C)** and instantaneous water use efficiency (WUE_inst_) **(D)** of drought-sensitive and drought-tolerant seed sources under control site(white bars; La Hunde site, considered the core habitat) and the dry site (black bars; La Granja d’Escarp considered the marginal dry habitat for this species). The letters above the bars marks the significant difference among the drought-stressed seed sources following the *post hoc* Duncan’s test with *n* = 5. Scale bars are mean +SE.

The maximal efficiency of PSII (*F*v/*F*m) was higher in both watered and drought conditions, but also differed significantly among drought-tolerant and drought-sensitive seedlings. Globally higher values were observed in drought-tolerant seed sources under the watered conditions, while this situation was slightly reversed after the observed decrease of PSII under drought stress (**Figure 1E**). Interestingly, the same pattern was observed regarding data obtained from the field (recorded during summer, see below); drought-sensitive seed sources showed higher PSII values than drought-tolerant ones under natural drought conditions at the Granja d’Escarp trial site (**Figures 1E, 2C**). The increase of quantum yield of non-cyclic electron transport (Φ_PSII_) showed slightly higher values in drought-sensitive seedlings than in drought-tolerant seedlings under drought stress (**Figure 1F**). Water use efficiency (WUE_inst_) was higher in the drought-tolerant seed sources under drought conditions (**Figure 1G**). This fact was consequence of greater capacity to maintain higher *P*n rates in drought tolerant seedlings for similar *E*-values in both, drought-sensitive and drought-tolerant.

We confirmed the validity of our experimental design by measuring the mentioned physiological parameters in plants of the same provenances grown in the field. Under field conditions the results obtained in the greenhouse regarding *E, P*n and WUE are in agreement with those observed in field under environmental conditions, given that the observed differences are not statistically significant (**Figures 1C,D,G, 2A,B,D**). However, *P*n followed the same trend with higher values in tolerant provenances and *F*v/*F*m values were also slightly lower, from 0.76 in sensitive seedlings to 0.75 in tolerant.

### Photosynthetic Pigments and Soluble Sugars

**FIGURE 3:**
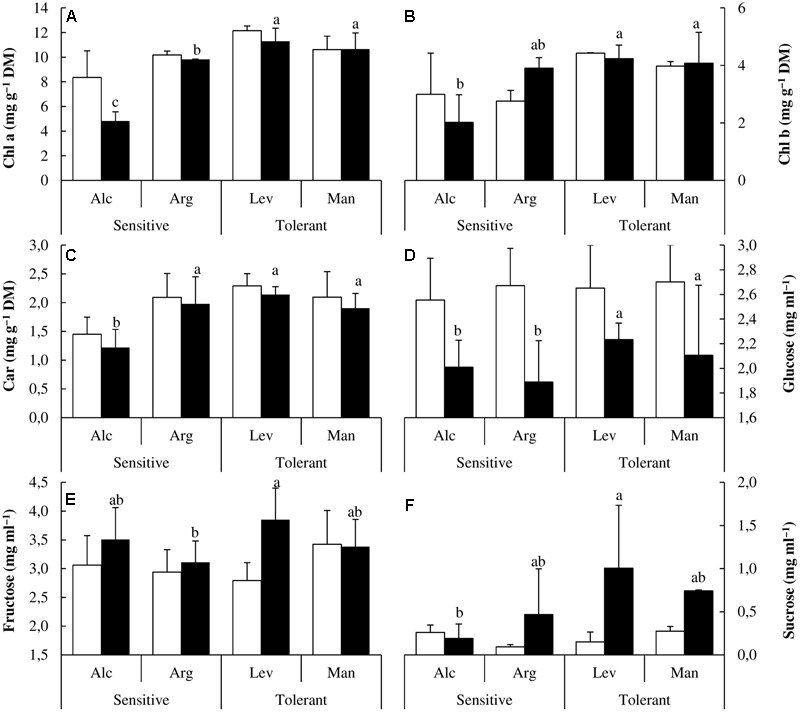
Photosynthetic pigments and soluble sugars content. Chlorophyll a (Chl a) **(A)**, chlorophyll b (Chl b) **(B)**, carotenoid (Car) **(C)**, glucose **(D)**, fructose **(E)** and sucrose **(F)** concentrations of drought-sensitive and drought-tolerant seed sources under watered (white bars) and drought stressed (black bars) treatments. The letters above the bars marks the significant difference among the drought-stressed seed sources following the test of Duncan with *n* = 5. Scale bars are mean +SE.

Once we confirmed the validity of our design, and observed the expected results regarding the physiological parameters related to water use and photosynthesis, we investigated the effect of drought on several photosynthetic pigments. Drought stress induced changes in photosynthetic pigments and differential patterns could be observed among drought-stressed seedlings from different origins. Chl a, Chl b and Car concentrations were less affected by drought in drought-tolerant seedlings and accumulation was 1.5- to 2-fold higher in drought-tolerant seed sources under drought conditions (**Figures 3A–C**).

One of the known physiological strategies to cope with drought stress is the osmotic adjustment through the accumulation of soluble sugars. In our samples, under drought conditions, the concentration of glucose decreased approximately 20 and 35% in drought-tolerant and drought-sensitive seed sources, respectively (**Figure 3D**). We could also observe differences in fructose and sucrose concentration. While fructose remains unchanged in the drought-tolerant seed source ‘Man,’ it increases about 40% in the drought-tolerant seed source ‘Lev.’ On the other hand it also increased by 20% in the drought-sensitive seed sources (**Figure 3E**). In addition, sucrose concentration increased about sevenfold in the drought-tolerant seed sources and only twofold in the drought-sensitive ones (**Figure 3F**) showing an enhanced accumulation in tolerant seed sources under conditions of water limitation.

### Glutathione and Free Amino Acids

**FIGURE 4:**
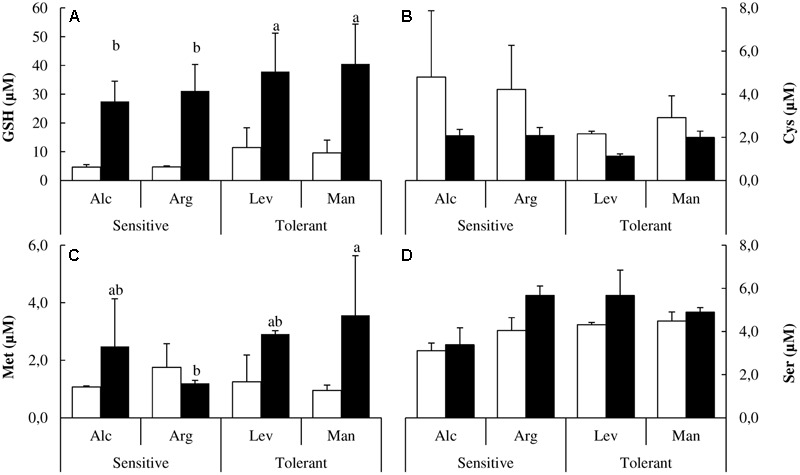
Glutathione, sulfur containing amino acids and serine determination. Glutathione (GSH) **(A)**, cysteine (Cys) **(B)**, methionine (Met) **(C)** and serine (Ser) **(D)** concentrations of drought-sensitive and drought-tolerant seed sources under watered (white bars) and drought stressed (black bars) treatments. The letters above the bars marks the significant difference among the drought-stressed seed sources following the test of Duncan with *n* = 5. Scale bars are mean +SE.

It has been shown that in response to drought stress the biosynthesis of sulfur containing amino acids may become limiting, specifically for the requirement of cysteine for the biosynthesis of glutathione (GSH), which is required to cope with the oxidative stress induced by drought (Mulet et al., 2004). In our study, GSH accumulated approximately 20% more in drought-tolerant seedlings upon drought stress (**Figure 4A**). Drought-tolerant seedlings contained less cysteine under control conditions but maintained the cysteine pool upon stress (**Figure 4B**). The increase of methionine (Met) concentrations under drought was higher than in sensitive seedlings (**Figure 4C**). Serine is required for cysteine and methionine biosynthesis. The concentration of this amino acid was higher in drought-tolerant seedlings both under watered and drought-stressed conditions (**Figure 4D**).

**FIGURE 5:**
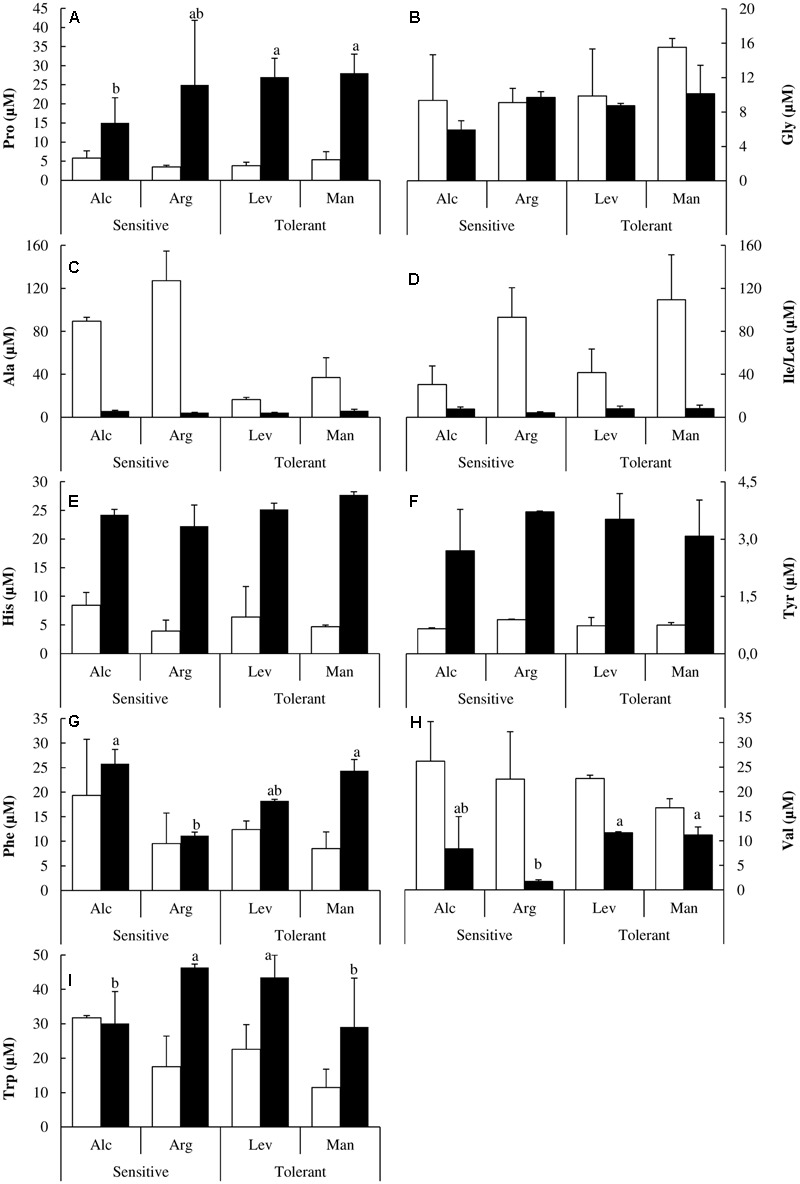
Non-polar amino acids and histidine determination. Proline (Pro) **(A)**, glycine (Gly) **(B)**, alanine (Ala) **(C)**, isoleucine/leucine (Ile/Leu) **(D)**, histidine (His) **(E)**, tyrosine (Tyr) **(F)**, phenylalanine (Phe) **(G)**, valine (Val) **(H)** and tryptophan (Trp) **(I)** concentrations of drought-sensitive and drought-tolerant seed sources under watered (white bars) and drought stressed (black bars) treatments. The letters above the bars marks the significant difference among the drought-stressed seed sources following the test of Duncan with *n* = 5. Scale bars are mean +SE.

There is no description available in the literature regarding the behavior of the free amino acid pools under drought stress in *Pinus halepensis*. Given that some of these amino acids can act as precursors for osmolytes, act as osmolytes themselves or even have previously undescribed functions in stress tolerance we investigated the complete free amino acid profile in our plants under the studied conditions. Proline and glycine are related to osmotic adjustment and, as expected, proline accumulated under drought stress. Interestingly this accumulation was higher in drought-tolerant seed sources (**Figure 5A**). Glycine concentrations were significantly higher in drought-tolerant seed sources under both watered and drought stressed conditions (**Figure 5B**).

Changes in the hydrophobic amino acids Alanine (Ala) and isoleucine/leucine (Ile/Leu) were also noteworthy; they decreased dramatically under drought stress. Alanine was higher in drought-sensitive seedlings while leucine/isoleucine was higher in drought-tolerant ones (**Figures 5C,D**). Histidine (His) and tyrosine (Tyr) concentrations were higher in the drought-sensitive seed sources under watered conditions but under drought stress, drought-tolerant seedlings accumulated higher concentrations (**Figures 5E,F**).

**FIGURE 6:**
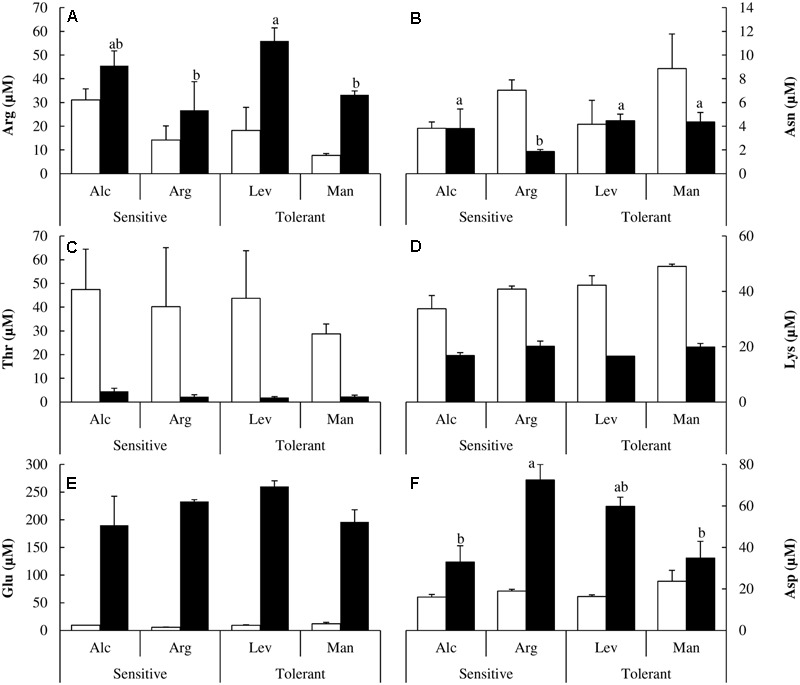
Polar or Charged amino acids determination. Arginine (Arg) **(A)**, asparagine (Asn) **(B)**, threonine (Thr) **(C)**, lysine/glutamine (Lys) **(D)**, glutamic acid (Glu) **(E)** and aspartic acid (Asp) **(F)** concentrations of drought-sensitive and drought-tolerant seed sources under watered (white bars) and drought stressed (black bars) treatments. The letters above the bars marks the significant difference among the drought-stressed seed sources following the test of Duncan with *n* = 5. Scale bars are mean +SE.

We did not observe differential behavior between drought-tolerant and drought-sensitive seed sources regarding charged amino acids (**Figure 6**). However, it is interesting to note how their concentrations change under drought stress. Arginine (Arg), glutamic acid (Glu) and aspartic acid (Asp) concentrations increased significantly (**Figures 6A,E,F**). The change for glutamic acid was about 20-fold (**Figure 6E**). Asparagine (Asn) and lysine/glutamine (Lys/Gln) concentrations decreased twofold (**Figures 6B,D**), and threonine decreased by more than 10-fold under drought stress (**Figure 6C**).

### Proteomic Analysis

Changes in the protein profile were also tested to determine which proteins potentially serve as biomarkers for stress tolerance or sensitivity, thus, facilitating the screening of tolerant seed sources in the future. We used 1D gel electrophoresis in order to perform side-by-side comparisons of eight different samples (four drought-sensitive and four drought-tolerant samples). We observed protein profiles of roots and needles under both watered and drought conditions. Under watered conditions we did not observe any significant change in the protein profile from needles. However, in roots under watered conditions we observed five differential bands in drought-sensitive and five in the drought-tolerant seed sources. The proteins identified from these differential bands were related mostly to carbon metabolism or protein translation (data not shown). These kinds of proteins are overrepresented in proteomes, independently of their biological origin, so these data were not considered to be relevant. Under drought stress, we observed the accumulation of proteins in the drought-resistant seed sources that are not present in the drought-sensitive seed sources. These results appear to be more informative since we found several heat shock proteins, a β-pinene synthase and the 5-methyltetrahydropteroyltriglutamate-homocysteinemethyl transferase (EC 2.1.1.14). This enzyme is responsible for the Vitamin-B-12 independent methionine biosynthesis, indicating that its function could be limiting under drought conditions (**Table 1**) and further confirming the increase in methionine in drought tolerant seedlings, as showed in **Figure 4C**.

**Table 1:** Proteomic analysis.

m/z	Score	NCBI/TrEmbl	Protein family
600,3	252	*Plectranthus scutellarioides Q42662*	5-methyltetrahydropteroyltriglutamate–homocysteine methyltransferase
636,8	127	*Oryza sativa* subsp. indica A2YWQ1	Heat shock protein 82
527,3	95	*Arabidopsis thaliana* P51818	Heat shock protein 81-3
804,4	93	*Zea mays* ACG43057	succinate dehydrogenase flavoprotein subunit, mitochondrial
630,3	166	*Citrus sinensis* XP_006488105	4-hydroxy-3-methylbut-2-en-1-yl diphosphate synthase, chloroplastic-like isoform X1
782,4	55	*Pinus radiate* AEW09360	hypothetical protein UMN_6182_01, partial
589,3	80	*Pinus banksiana* AFU73844	(-)-beta-pinene synthase
950,2	98	*Picea sitchensis* ACN40706	Unknown (∼amidase 1-like)
837,4	102	*Picea sitchensis* ABK23116	Unknown (∼chloroplast photosystem I subunit)
472,8	83	*Picea sitchensis* ABK27024	Unknown (∼histone H2A)

*Proteins of differential expression identified among roots of *Pinus halepensis* plants tested under drought-stressed conditions.*

## Discussion

*Pinus halepensis*, commonly known as Aleppo pine is a useful plant for reforestation due to its ability to adapt to fluctuations in temperature and soil water availability during its life cycle (Baquedano and Castillo, 2006; Baquedano et al., 2008). Here we have compared at the molecular and physiological level the effect of drought in different seed provenances.

Throughout this study, the water potential (Ψ_w_) exhibited significant reductions in drought-stressed *Pinus halepensis* seedlings, thus, validating our experimental design and confirming that the seedlings in the greenhouse were affected by the drought conditions employed. It is known that one of the main problems caused by drought is oxidative damage (Golldack et al., 2014) affecting several plant processes. One of the strategies to avoid this constraint is to regulate the energy status (Voss et al., 2013) which suggests that drought-tolerant seedlings are more efficient at down-regulating the maximum efficiency of PSII and thus avoiding oxidative stress. Taken together, our data indicate that the most important parameter for drought tolerance is the ability to reduce the efficiency of photosystem II. A recent report has shown that a similar behavior has been observed in other plants, specifically a higher photosynthetic rate in the favorable season was associated with a stronger decline in the unfavorable season (Zhang et al., 2017), which would be similar to what we are observing here for *P. halepensis*. Drought tolerant plants also accumulate more photosynthetic pigments than drought sensitive plants (**Figures 3A–C**) likely diminishing photo-oxidative damage and ROS production (Vilagrosa et al., 2010). In this sense, the pattern of response in the present study in relation to gas exchange and photochemical efficiencies under control and drought conditions follows the results observed in previous studies although our data are located in the lower range of observed values (Baquedano and Castillo, 2006; Cuesta et al., 2010; Klein et al., 2012). This is probably consequence of seedling age as we were using very young seedlings (<1 year) and the water saving strategy followed by this species in small seedlings increases water economy (Baquedano and Castillo, 2006). This effect has been already observed when analyze gas exchange patterns in some Mediterranean species with moderate gas exchange rates (Vilagrosa et al., 2003; Atzmon et al., 2004; Ruiz-Yanetti et al., 2016) and partially can be attributed to xylem characteristics with short and narrow vessels (Vilagrosa, pers. obs). In addition, several studies analyzing plant provenances highlighted the relevance of morphological and functional factors allowing plant acclimation to stress conditions, from hydraulic effects (Peguero-Pina et al., 2014) to changes in mesophyll conductance affecting to photosynthetic parameters (Peguero-Pina et al., 2017).

We extended our research to study the NSC which are among the most drought-responsive metabolites. Their concentrations could increase due to starch hydrolysis or impairment of starch production (Rodríguez-Calcerrada et al., 2011). In *Pinus halepensis* the most distinctive feature of drought-tolerant seed sources was the increase in both sucrose and fructose. Sucrose can act as an osmolyte to prevent water loss and protect cellular structures from dehydration. Therefore; the ability to accumulate sucrose under drought stress could be another key factor to distinguish between drought-tolerant and drought-sensitive seed sources (**Figure 3F**).

Under drought stress, seedlings can down-regulate photosynthesis, accumulate osmolytes or improve the antioxidant response to prevent the deleterious effects caused by ROS. The synthesis of cysteine from serine and the subsequent biosynthesis of GSH is a key aspect of antioxidant defense, especially the activity of the serine acetyl transferase enzyme, which may be considered as the main limiting factor (Freeman et al., 2004; Mulet et al., 2004). Recent reports confirm that GSH is considered to be the most important thiol involved in the prevention of oxidative damage in plants (Labudda and Safiul-Azam, 2013; Pyngrope et al., 2013). We could confirm that GSH accumulation is also a key and a distinctive factor for drought tolerance in woody plants as the drought-tolerant seed sources present a fourfold increase in response to drought stress (**Figure 4A**).

There are several reports in the literature illustrating how the levels of different amino acids change under drought stress. Here, we have performed a complete analysis of free amino acids. This is the first study at the amino acid level comparing different *Pinus halepensis* seed sources under drought stress. We found a distinctive pattern in sulfur amino acids. Drought-tolerant seed sources have less Cys and less Met than the drought-sensitive ones under watered conditions, but they maintain their levels of Cys and increase those of Met under drought stress. However, the drought-sensitive seed sources decrease Cys levels and maintain the levels of Met under drought stress (**Figures 4B,C**). Ser levels remain unchanged under stress, but they are higher in the drought-tolerant seed sources. The complete analysis of free amino acids pools under drought stress showed significant increases in the content of Tyr, His, Pro, Arg, Glu and Asp along with a decrease in Ala, Ile, Val, Thr and Lys concentrations (**Figures 4, 5**). The increase in Pro (approximately sixfold; **Figure 5A**) can be explained because of its activity as an osmolyte (Serrano et al., 1999; Verbruggen and Hermans, 2008); the Gly content was higher in the drought-tolerant seedlings, but the levels drop under stress (**Figure 5B**), probably due the increase of GSH. The interpretation of the remaining changes goes beyond the scope of this article.

The proteomic study also generated valuable information. Under watered conditions we did not observe any differential bands in needles, but several proteins were differentially expressed in roots. As expected, most of the identified proteins are related to the transcriptional machinery and sugar metabolism. Because proteins participating in these processes are over-represented in proteomes, we considered them irrelevant for the purpose of this study (data not shown). More significant results were obtained in drought stressed roots (**Table 1**) where we detected two different heat shock proteins of the 82 and 81-3 type. The role of these proteins is to act as molecular chaperones that promote the maturation, structural maintenance and proper regulation of specific target proteins in an ATP-dependent manner. Under drought stress, misfolded proteins are likely to accumulate, therefore, the increase in this kind of proteins is surely beneficial for defense against cellular stress. We also found a β-pinene synthase to be up-regulated in roots under drought stress conditions. This protein is regulated in a circadian manner. Given that all samples were collected at the same time and that its enzymatic activity is exerted mainly in leaves, we cannot discard an undescribed role for this enzyme in stress response in roots.

Another interesting finding is the accumulation of the 5-methyltetrahydropteroyltriglutamate-homocysteine methyltransferase (EC 2.1.1.14) in the roots of drought-tolerant seedlings. This enzyme is responsible for the Vitamin-B12-independent methionine biosynthesis. In bacteria, this pathway has been related to abiotic stress response (Mordukhova and Pan, 2013). It is known that this pathway exists in plants (Eichel et al., 1995). This enzyme was identified in a proteomic study as being important for somatic embryogenesis in *Quercus suber* (Gomez-Garay et al., 2013). However, its relation to drought stress has not been postulated before.

Here we report that under drought stress the drought-tolerant seedlings increase the production of Met. As a result, it is possible that the alternative pathway for Met biosynthesis is more active in the drought-tolerant seedlings leading to the increase of Met supply. Met is a substrate for the synthesis of various polyamines with important roles in drought tolerance (Groppa and Benavides, 2008; Alcázar et al., 2010). Sulfur amino-acids metabolism is determinant for drought stress tolerance although the enzyme which becomes limiting could change in different species (Freeman et al., 2004; Mulet et al., 2004). Our results indicate that the alternative biosynthesis of Met, together with the synthesis of GSH are associated to drought tolerance in *Pinus halepensis*.

## Conclusion

We have used a Greenhouse-based approach to determine differential traits at the physiological and molecular levels between drought-tolerant and drought-sensitive seed sources of *Pinus halepensis*. Our results indicate that the most distinctive trait for drought tolerance in *Pinus halepensis* seedlings are related to oxidative stress. Drought-tolerant seed sources presented a more pronounced down-regulation of PSII and a higher accumulation of photosynthetic pigments including carotenoids which act mainly as antioxidants. In addition, we propose that the photosynthesis rate and the maximal efficiency of PSII could constitute potential distinctive traits among drought-tolerant and drought-sensitive *Pinus halepensis* seedlings.

Analysis of free amino acids indicated that there was a significant increase of GSH and Met concentrations. The increase in Met was corroborated at a different level by the identification of a Met synthase as a differential protein band present in stressed drought-tolerant seed sources.

Taken all together, we propose that the analysis of photosynthetic parameters (photosynthesis rate, the maximal efficiency of PSII and photosynthetic pigments), along with the determination of fructose, Met and GSH concentrations could be a fast and reliable method to screen for *Pinus halepensis* seed sources which are more likely to be drought tolerant.

Our findings are the basis to develop valuable evaluation tools to determine the appropriate seed source for reforestation programs threatened by drought, thus avoiding long field trial tests.

## Author Contributions

KT planned, performed experiments and wrote the paper. AV assisted in the photosynthetic gas exchange and chlorophyll fluorescence measurements. JB and ML-G helped with the sugar content analysis. DP and JC performed the mass spectrometry identification of the proteins. JL-N undertook the free amino acid analysis. AdC and JM planned, guided and performed experiments and wrote the paper.

## Conflict of Interest Statement

The authors declare that the research was conducted in the absence of any commercial or financial relationships that could be construed as a potential conflict of interest.
